# Supplementary material for: Are carbon emissions trading and green financial instruments synergistic? -Comprehensive quantitative research based on content analysis
Source: PLoS One. 2024 Mar 7;19(3):e0298601. doi: 10.1371/journal.pone.0298601 (PMC10919601; doi:10.1371/journal.pone.0298601)
Supplement: S1 Data — (ZIP) [file pone.0298601.s001.zip › Data/地级市Z_F工作报告.zip!/╣π╢1⁄2/╔╟═╖╩╨/└┤╫╘╣1⁄2╓┌║┼í╛┬φ┐╦╩2╛▌═°í┐_2015.docx]

各位代表：

我代表汕头市人民政府，向大会报告工作，请予审议，并请各位政协委员和其他列席人员提出意见。

一、2014年工作回顾

过去一年，面对复杂多变的国内外经济形势，市政府在市委的正确领导和市人大、市政协的监督支持下，紧紧围绕省委、省政府关于促进粤东西北地区振兴发展的决策部署，以交通基础设施建设、产业园区扩能增效、中心城区扩容提质为抓手，真抓实干，奋力拼搏，在加快汕头振兴发展的征程中迈出坚实的步伐。

（一）经济保持平稳增长。认真落实国家和省稳增长的各项政策，出台扩投资、促消费、稳外贸等一系列具体措施，有力有效应对全国经济下行压力，确保全市经济发展保持稳中有进、稳中提质的良好势头。全市实现生产总值1716亿元，增长9％，高于全国、全省增长水平；一般公共预算收入123．97亿元，增长10．6％，超额完成年度预算目标，实现规模和质量双提升。积极争取上级公共预算转移支付补助101．35亿元，比上年增加19．58亿元。固定资产投资延续高位增长态势，首次突破千亿元大关，完成1002．73亿元，增长35．1％，增速列全省第五，制造业投资463．2亿元，增长37．7％，占总量46％，投资对实体经济的拉动作用进一步显现。消费市场保持畅旺，实现社会消费品零售总额1286．04亿元，规模稳居全省前五，电子商务、信息消费成为新的消费热点，快递业务量在全国排第39位、全省第六位、粤东西北第一位。外贸进出口总额95．6亿美元，增长3．5％，高于全国、全省增长水平。金融机构本外币存款余额2664．46亿元，总量居全省第六，贷款余额首次突破千亿元大关。工业用电量、港口货物吞吐量、集装箱吞吐量、旅客周转量等先行指标保持向好，经济持续平稳增长的基础比较牢固。

（二）交通基础设施建设成效初显。集中资源和力量，攻坚克难，破解资金和征地拆迁难题，交通基础设施建设大会战如火如荼。汕头境内五条高速公路建设节节推进，汕揭高速公路建成通车并与沈海高速互联互通，潮惠、揭惠高速公路分别完成总投资量32％和31％，汕湛高速公路推进控制性工程建设，潮汕环线高速公路前期工作进展顺利。全长11．08公里的南澳大桥建成通车，打通了南澳连接大陆的快速通道，为南澳打造成为国际高端旅游休闲岛创造条件，也成为一道亮丽的风景。汕头海湾隧道工程初步设计方案已获省交通厅正式批复，即将启动建设。铁路建设取得重大突破，社会各界和人民群众热切期盼的广梅汕铁路增建二线及厦深铁路进汕头联络线工程启动施工，汕头港疏港铁路前期工作进展顺利，市域轨道交通完成初步规划。干线公路升级改造全面提速，国道324线潮南新庆至洋汾陈路段、省道231线澄海凤湾路建成通车，国道324线潮阳梅花至新庆路段和省道237线潮南和惠路单侧通车，国道324线峡山路段改线工程和省道337广葵线磊广路、河浦大道、省道235司神线建设加快推进，新开工国道206线二期、国道324线莲阳桥至盐鸿段、省道234揭海线等35．9公里路面大修工程。广澳港区防波堤工程加快建设，10万吨级航道工程启动项目前期工作，两个10万吨级集装箱泊位工程初步设计获交通运输部批复。华能海门煤炭中转基地主体工程建成投产。云澳国家级中心渔港建成投入使用。

（三）产业转型升级势头良好。实施“四大产业计划”和“五个100工程”，出台培育大型骨干企业、新一轮工业企业技术改造等政策措施，助推产业转型升级。促进传统产业高端化，完成工业技改投资187．69亿元，增长68．2％，总量居全省第二。新认定“中国驰名商标”7件、“广东省著名商标”9件，新认定省高新技术产品155项，新增省名牌产品31个，新增省级工程技术研究中心14家，新增专利申请9097件、专利授权6470件、PCT国际专利申请26件。金平区建成中小企业综合服务窗口、工业成果展览厅、知识产权服务中心、食品及装备研究院、轻工装备研究院等公共服务平台。澄海区被确定为“国家知识产权强县工程试点区”、“全国产业集群区域品牌建设玩具产业试点地区”。现代产业加快发展。广东移动粤东区域生产中心一期竣工，汕头世纪互联大数据协同创新产业园开工建设，“中国锆城”核用条带、复合氧化锆、锆钛精选项目竣工投产。广澳物流园建设加速推进，宝奥城正大粤东配送中心正式开业、货运超市基本建成。长平新一城投入运营，广厦综合体竣工，苏宁广场、百脑汇生活广场、华润万象城、华银商业综合体、澄海猛狮国际广场、潮南巴比伦商贸城和国瑞会议会展中心、雅士利总部大楼加快建设，城市现代化气息逐步显现。电子商务发展迅猛，入选全国电商百佳城市，网商密度居全国第23位，全市近90％的企业开展电子商务应用，交易规模连续四年增速超过50％；现有“淘宝村”10个，数量在全国名列前茅，在全省排第二位。金融业稳健发展，新增金融机构6家，全省首家科技金融服务中心挂牌运作，在全省率先成立“新三板”服务中心，在“新三板”挂牌企业6家。农业现代化建设取得新突破，澄海区获省推荐申报国家级现代农业示范区，潮阳区完成农业现代示范区总体规划编制工作，南澳县成为第一批省级新农村建设示范片，新增农民专业合作社109家，累计达到900家。文化创意产业加快发展，全市有11家企业投资超过60亿元进军动漫、影视、手机游戏等领域，粤东首个文化创意园——1860文化创意园正式开园。实施旅游兴市战略，澄海前美古村侨文化旅游区成为AAAA级旅游景区，南澳青澳湾成为国家级海洋公园，潮商旅游休闲游艇码头、绿梦湿地生态园、锦峰动感世界等一批旅游休闲项目加快推进，区域旅游休闲中心地位凸显。

（四）战略平台实现重大突破。汇聚海内外潮人力量，积极争取国家支持，在不到一年的时间内，国务院就批复同意在汕头经济特区设立华侨经济文化合作试验区，汕头的发展再次上升为国家战略，世界的目光再次聚焦汕头。试验区管理委员会正式揭牌成立，搭建扁平化、高效率的管理体制及运作机制。试验区总体规划已委托中国国际经济交流中心编制，基础设施、产业引进等专项规划编制同步进行。政策创新研究有序开展，梳理形成华侨试验区拟争取政策目录，涉及金融、贸易投资、人员往来便利化等7个领域。招商引资工作积极开展，一批旅游休闲、文化创意、金融服务等投资项目落户试验区。起步区加快建设，东海岸新城累计投资近100亿元，围填海及水利主体工程全面完成，中山东路东延、滨江大道基本建成，新津河、外砂河大桥加快建设。珠港新城总部基地总部大楼项目加快推进。南滨新城土地一级开发、潮汕历史文化博览中心及一批市政基础设施、生态景观工程进展顺利。粤东物流总部新城土地一级开发和泰山路北延工程加快推进，西部生态智慧新城、潮阳东部新城、潮南行政新区、厦深高铁潮阳站（谷饶）物流新城等区域平台全面推进。

（五）城乡环境明显改善。有序推进城市更新，加快小公园开埠历史文化街区、潮人码头建设，改造外马路、中山路、跃进路、长江路等共10万平方米破旧道路和一批内街小巷，以及澄海、濠江、潮阳、潮南、南澳城区一大批市政道路，城乡面貌大为改善。推动城市管理重心下移，构建网格化管理模式，推行环卫作业市场化，市容环境卫生有所改观。深入开展城市管理联合执法，集中整治占道经营、非法营运、乱停乱放等违法行为，城市更加规范有序。着力加强生态环境建设，敢于直面解决城乡多年累积的严峻生态问题。澄海洁源环保发电厂试运营，市雷打石环保电厂、雷打石生活垃圾卫生填埋场扩建工程、潮阳区垃圾焚烧发电厂、潮南区垃圾焚烧发电厂和一批污水处理厂建设取得突破性进展。练江流域综合整治纳入省委、省政府重要议事日程，省安排水环境综合治理资金3．5亿元，环保、水利规划和纺织工业循环经济产业园区潮南片区规划编制完成。贵屿循环经济产业园区建设加快推进，污水处理厂、垃圾填埋场、危险废物转运站和电子废物集中拆解楼前三期建成投入使用，湿法、火法冶炼项目正式动工，环境修复试点工作稳步推进。加强水利基础设施建设，汕头大围主体工程基本完成，“百河千沟万渠大整治”行动和潮阳、潮南“村村通自来水”工程建设进展顺利。继续开展“绿满家园”全民行动，启动国家森林城市创建工作。深入开展“平安汕头”建设，扎实推进“社会矛盾化解年”工作，大力开展“三类重点地区”整治，全市刑事、治安警情分别下降9．3％、8．3％，交通事故死亡人数连续九年呈下降趋势，人民群众安全感不断提升。落实“党政同责、一岗双责、齐抓共管”机制，狠抓安全生产隐患排查治理，未发生较大以上生产安全事故，社会平安和谐。国防建设和民兵预备役工作得到加强，双拥工作深入推进，军政军民更加团结。

（六）改革开放不断深化。扎实推进行政审批制度、商事登记制度、企业投资体制等有利于激活市场活力的改革。本轮行政审批制度改革取消、转移、下放、前移政府职能事项456项，调整比例61．5％；完善228项事项的办事依据，优化231个审批环节，压缩56项事项的办事时限，减少232个审批工作日。商事登记实行“先照后证”、注册资本认缴登记制，对涉及市场主体经营项目许可的108项前置审批改为后置审批，全年新登记企业5109家，注册资本145亿元，分别增长39．2％和76．7％。不断简化企业投资管理流程，取消市级权限内企业投资项目核准，建立以备案制为主的企业投资项目管理体制。建立中心城区税收属地征管财税利益调节机制，构建全口径政府预算体系，积极推进预算信息公开。理顺工商、质监、卫生部门的食品药品监管职能，统一归口食品药品监督管理局，市场监管体系进一步完善。创新人才引进机制，出台“1＋10”人才奖励方案，最高予以500万元奖励。推进民政工作改革创新，在全国地级市率先出台募捐条例、村务公开条例和社会工作者条例，民政法制建设经验在全国得到推广。推动更高层次的“引进来”、“走出去”。成功举办海峡两岸海洋经济合作交流会，达成合作项目100多宗，总金额167．3亿元。南澳获批设立海峡两岸交流基地。新增境外投资额6．72亿美元，增长18．2倍，在南非设立“广东品牌联盟展销中心”，在阿联酋迪拜设立“中东—中国手机批发城”。推广省内海关区域通关一体化改革，全面推进关检“一次申报、一次查验、一次放行”模式和通关无纸化改革，建立新型企业进出口信用管理制度，实施出口预检制度，通关效率大幅提升。汕头保税区申报设立保税物流中心（B型），向综合保税区转型。

（七）社会民生逐步改善。优先保障民生事业公共财政支出，全年共支出141．48亿元，比上一年增加12．5亿元。新增城镇就业6．1万人，农村劳动力转移就业5．33万人，就业形势保持稳定。全面推进教育创强，全市现有金平区、龙湖区两个教育强区和17个教育强镇（街道），澄海区、南澳县申请省教育强区（县）验收。义务教育标准化学校覆盖率达92．5％，金平区、龙湖区通过全国义务教育基本均衡区督导评估。教育质量继续提升，普通高考总上线率达91．7％，比全省高出8个百分点。广东以色列理工学院筹备工作全面推进。市高级技工学校新校区建成并按期招生。加大医疗卫生投入，全市新增床位560张，每个区（县）均设立一所“平价医院”，边远乡镇卫生院医务人员津补贴人均月增加500元。下大力气解决市中医院、市妇幼保健院、市皮肤医院、汕大国际眼科中心异地建设用地问题，促进医疗资源布局更加均衡。加大社会办医力度，金平太安医院等一批民营医疗机构建成开业，汕头东方医院等6家民营医院加快建设，潮南民生医院成为粤东首家民营三级医院。加强人口计生工作，稳定低生育水平。全面推进社保扩面征缴工作，全市参加企业职工养老保险118．4万人、医疗保险50．1万人，城乡居民社会养老保险189．4万人、基本医疗保险455．1万人。公共文化设施建设不断完善，达标升级建设10个综合文化站、300个行政村（社区）文化室，完成5个镇（街道）、130个行政村（社区）公共电子阅览室的硬件设备配套建设；市非物质文化遗产展示馆基本建成，潮阳博物馆建成开放，潮南博物馆、文化馆、图书馆挂牌运作，区（县）一级数字影院建设实现全覆盖。龙湖区荣获“全国文化先进区”称号，澄海区通过“全国文化先进区”复查验收。精品剧目《古城风雷》、《大潮商魂》分获省艺术节“优秀剧目一等奖”和“优秀剧目奖”。启动“海上丝绸之路”文物史迹申报世界文化遗产工作，汕头抽纱、贵屿双忠信俗、陶瓷微书、太安堂中药文化等4个项目入选第四批国家级非遗名录。加大公共交通投入，新投放公交车153辆，其中新能源（LNG）公交车43辆，调整、新增一批公交线路。深入开展全民健身活动，推动乡镇农民健身工程建设，新增健身广场71个。加大底线民生保障力度，社会救助水平稳步提高。推动多元化养老服务体系建设，实施“银龄安康行动”，启动长者紧急呼援服务，为全市80周岁以上老人和60至79周岁的五保低保老人统一购买意外伤害综合保险，80周岁以上老人享受高龄津贴。通过“全国白内障无障碍市”验收，救助残疾人超过5万人次。全面完成汕潮揭医保同城化联网结算，全市异地就医联网医疗机构达22家。住房公积金在汕潮揭三市实现缴存互认、跨市互贷。加快推进殡葬服务均等化进程，全市普惠型殡葬惠民政策覆盖人群超过270万人。积极构建公共法律服务体系，推进潮阳、潮南区一村（社区）一法律顾问试点工作。扶贫开发“双到”取得新成效，筹措帮扶资金近2亿元，建设帮扶项目1113个。民族宗教领域和谐稳定，81处宗教活动场所获得全国“和谐寺观教堂”称号。去年确定的十大民生实事完成年度任务（见附件1）。

（八）政务环境持续优化。推进依法行政，打造阳光法治服务政府。坚持向市人大及其常委会报告工作，向政协通报情况，自觉接受监督。办理人大代表议案（建议）63件，政协提案183件。充分运用特区立法权，提请市人大常委会审议法规草案　4件，制定政府规章　13　件。推进行政复议委员会试点工作，受理复议案件146件，办结123件。扎实推进普法工作，我市被评为全国“六五”普法中期先进城市。在全国率先出台制度廉洁性评估办法，　从制度层面加强和规范政府自身建设。基本完成市县政府机构改革任务，形成权责明晰、衔接有序、配合有力的部门间工作协调机制。加快电子政务建设，全面启动“汕头政府在线”项目，完成59家单位办公自动化系统建设，45个市级单位766项行政审批和便民服务事项梳理上网，实现政务办公、行政审批、公众服务、效能监察的全流程电子化。整合64个单位71条政务服务热线，打造集咨询、投诉、举报为一体的政府12345便民服务热线平台。落实监察、审计部门负责人固定列席市政府常务会议制度，全程介入政府决策的酝酿、讨论、决定、实施等环节，促进行政管理与行政监察、审计监督同步。深入开展党的群众路线教育实践活动，认真落实中央八项规定，有力抓好“四风”整改，修订完善行政事业单位会议费、差旅费等管理办法，建立厉行节约长效机制，会议费及“三公”经费下降　37％；大力精减文山会海，市政府全市性会议减少28％，以市政府及办公室名义下发文件减少23％，政府作风明显改进，行政效能明显提升，政务环境逐步改善。

各位代表！过去一年我们所取得的成绩和进步，是省委、省政府和市委正确领导的结果，是全市人民同心协力、共同奋斗的结果，是海内外乡亲大力支持、积极参与的结果。在此，我代表市人民政府，向全市广大工人、农民、知识分子、干部职工，向给予我们支持的人大代表、政协委员、各民主党派、工商联、社会各界人士和海内外乡亲，向中央和省驻汕单位，向驻汕解放军、武警官兵，向所有关心和支持汕头发展的港澳台同胞、海外侨胞和国际友人表示崇高的敬意和衷心的感谢！

我们工作中还存在许多不足，经济社会发展还累积很多亟待解决的矛盾和问题：一是经济持续较快增长的动力和后劲不足，传统产业转型升级压力大，现代产业尚未形成新的增长点；二是改善民生任重道远，教育、医疗、社会保障等公共事业发展相对滞后，民生保障还不到位，与人民群众的需求差距仍然较大；三是社会管理水平亟须提高，交通秩序、市容市貌、生态环境还没有根本性改观，群众反映依然强烈；四是推进依法行政、建设阳光法治服务政府还存在不少薄弱环节，政府服务意识、服务水平、办事效率还有很大的提升空间。我们必须正视自身工作中存在的问题，采取有效措施认真解决。

二、2015年工作安排

新的一年，是“十二五”规划的收官之年。今年政府工作的总体要求是：全面贯彻党的十八届四中全会、省委十一届四次全会精神，按照市委十届五次全会的部署要求，坚持稳中求进工作总基调，坚持加快发展和提高质量并重，认真落实省委、省政府促进粤东西北地区振兴发展的一系列政策措施，主动适应经济发展新常态，深化改革开放，突出创新驱动，做大产业龙头，统筹城乡发展，加强民生保障，推动经济社会发展迈上新台阶。

今年经济社会发展的主要预期目标是：地区生产总值增长9％，固定资产投资额增长20％，进出口总额增长8％，社会消费品零售总额增长12％，一般公共预算收入增长10％，居民消费价格指数涨幅控制在3％左右，居民收入增长与经济增长基本同步，城镇登记失业率控制在3％以内，节能减排约束性指标完成省下达年度计划。需要说明的是，今年地区生产总值预期增长目标确定为9％，高于省的预期增长目标（7．5％左右），主要是贯彻落实省委、省政府促进粤东西北地区加快发展、振兴发展的要求，必须自加压力、赶超进位；同时也是考虑经过这几年的努力，我们初步夯实了加快发展的基础，稳增长的态势仍将持续。但要完成这样的目标任务，我们还是要付出艰辛的努力。

按照上述目标任务，今年要重点抓好七方面工作：

（一）乘势而上打好交通基础设施建设攻坚战

我市交通基础设施建设大会战已进入攻坚阶段，我们要乘势而上，攻坚克难，加快打通对内对外快速通道，全力构建互联互通的现代交通网络，对接辐射闽粤赣传统腹地，拓展经济发展空间。

高速公路方面。加快潮惠、揭惠高速公路建设进度，全线铺开汕湛高速公路建设，新开工建设潮汕环线高速公路。

干线公路方面。开工建设汕头海湾隧道主体工程。加快国道206线大学路二期工程、国道324线峡山路段改线一期工程、国道324线莲阳桥至盐鸿段等9个在建项目建设，开工建设金凤路桥潮汕路至大学路高架桥工程，启动国道324线潮南洋汾陈至石桥头路段、省道336线南澳环岛公路、省道237线和平至贵屿段、省道335线澄海路段、省道337线河浦至海门路段、省道234线棉城新宫至海门段路面大修工程。加快牛田洋通道规划研究和国道324线礐石大桥至红旗岭路段、省道336线莱美路改造工程等项目前期工作。

铁路方面。加快推进广梅汕铁路增建二线及厦深铁路进汕头联络线工程建设，规划服务粤东地区的铁路口岸，加快汕头港疏港铁路、市域轨道和粤东城际轨道交通项目前期工作。

港口方面。开工建设广澳港区二期两个10万吨级集装箱码头和2万吨级石化码头。加快广澳防波堤、华能海门煤炭中转基地工程建设，继续推进汕头港广澳港区航道二期工程前期工作。

（二）以华侨试验区建设引领新一轮改革开放

紧跟时代改革创新步伐，直面社会各界和群众期盼，以建设华侨试验区为契机，推进重点领域和关键环节改革，倒逼和引领汕头体制机制创新，全面提升开放型、服务型政府建设水平，进一步激发市场活力和社会资本内生动力。

深化行政管理体制改革。创新华侨经济文化合作试验区体制，实施权力清单、责任清单、负面清单制度。建立符合广大海外华侨华人意愿和国际通行规则的跨境投资和贸易规则体系，促进贸易和投资便利化。加强金融、贸易投资、人员往来便利化、现代服务业和新兴产业、用地用海、文化教育医疗、民政和财税等方面政策创新，积极争取国家和省的政策支持。进一步简政放权，加大行政审批事项取消、调整力度，全面推进清理行政职权和编制权责清单工作，公布部门职权目录，为企业打造“一口受理、并联审批、保姆式服务”的高效政务服务模式，推动政府职能转变取得新突破。加快建立健全与行政审批制度改革相配套的后续监管制度体系。深化乡镇行政体制改革，推进管理服务职能重心下沉。加大政府购买公共服务力度，出台适合汕头实际的政府购买公共服务实施项目。制定《汕头市电子政务建设管理办法》，规范全市电子政务建设管理工作，完成“汕头政府在线”全面覆盖期建设任务，推动“汕头政府在线”在各区县的推广应用；继续拓展完善市、区（县）网上办事分厅和实体大厅建设，推进向村居延伸。

深化商事登记制度改革。全面推行“先照后证”、注册资本认缴、商事登记联办、注册登记网上验资、市场主体年报备案制度，加快建立“宽进严管”的市场准入体系。加快探索推行工商营业执照、组织机构代码证和税务登记证“三证合一”登记制度，探索企业注册“一表通”机制，推动电子证照系统建设，进一步方便企业和群众办事。加强商事制度改革工作立法。全面推进社会信用体系和市场监管体系建设，加快构建市场主体信用信息公示系统，严格执行经营异常名录和严重违法企业名单制度，强化信用约束。通过放管结合、放管并重，充分释放市场活力，激发大众创业、万众创新的动力。

深化金融改革创新。谋划在试验区设立“华侨银行”、“华侨股权投资基金”。争取亚投行、丝路基金在试验区设立分支机构，拓展离岸市场人民币资金回流渠道。大力发展股权（创业）投资基金，推动设立期货产品现贷交割地和大潮汕股权交易中心。积极发展互联网金融、新型电子支付业务。推进汕特农信联社改革，加快澄海、潮阳、南澳农信联社改制为农商行。开展农村普惠金融试点工作。

深化土地管理制度改革。探索用海用地衔接机制，积极争取东海岸新城围填海新增建设用地不占用既有建设用地规模指标。建立城乡统一的建设用地市场，在符合规划和用途管制前提下，允许农村集体经营性建设用地出让、租赁、入股，实行与国有土地同等入市、同权同价。创新特区土地规划管理体系，完善公益性项目建设用地优先保障机制，以濠江区、潮阳区为试点推行相对集中建设农民公寓住宅。积极稳妥推进农村土地承包经营权确权登记工作。建立不动产统一登记制度。

全面提升开放型经济水平。围绕试验区跨境金融、文化创意等九大产业，加大对外开放和招商引资力度，推动一批产业项目落地建设。主动对接国家“一带一路”战略，争取在试验区设立“中国侨商大会”、“中国华侨博览会”等具有国际影响力的永久会址。加强与海上丝绸之路沿线重要城市交流合作，推动各国侨商会在试验区设立办事处、基金会，依托潮人社团、商会设立一批海外经贸联络处。加强与海内外潮人商会合作，共同谋划建设华侨产业新城，争取设立“华侨华人产业交易会”，打造华人华侨产业交易博览会大型永久展览场馆。推动进出口协调发展，发挥促进进口专项资金引导作用，扩大鼓励进口贴息范围，积极扩大先进技术、关键装备进口；推动外贸转型升级示范基地建设，加强出口自主品牌培育，推进区域品牌境外注册，稳定外贸出口。借力侨商侨团作用，积极为企业“走出去”搭建平台，支持企业开展跨国并购，建设境外营销网络，参与境外基础设施工程承包、能源资源合作开发。

（三）扎实推动产业转型升级

继续实施“四大产业计划”和“五个100”工程，以培育大型骨干企业为龙头，以自主创新和技术改造为抓手，以发展现代产业为突破口，加快打造粤东西北产业转型升级新亮点。

大力培育战略性新兴产业。重点做强做大锆产业、大数据产业、北斗导航和卫星遥感产业、环境友好型新材料产业等四大产业。锆产业方面，落实省市共建锆产业基地产业链条项目，争取在锆产业关键领域实现重点突破。大数据产业方面，依托国际海缆登陆站及用电资源优势，加快推动民营海缆登陆站运营和智慧能源岛项目建设。加快大数据协同创新产业园建设，吸引一批新一代信息技术企业落户。加快建设互联网生活创业小镇，吸引IT人才落户。北斗导航和卫星遥感产业方面，全面开展信息消费试点城市建设，突出行业特点，重点推广面向传统优势产业转型升级的应用。环境友好型新材料产业方面，加快省市共建可降解环保产业基地规划建设，推动益德环保100万吨全生物降解农地膜项目投产。推动中核集团新材料、超声电子、乐凯集团锂离子电池隔膜等一批项目落地。

加快发展现代服务业。优先发展金融业，积极引进港澳台及境内各类金融机构入驻；利用多层次资本市场，全面提升“金融超市”和“新三板”服务中心功能，争取8家IPO在审企业尽快上市，缓解中小企业融资难。大力发展都市型商贸物流业，加快推进华润万象城、苏宁广场、百脑汇生活广场、澄海猛狮国际广场、潮阳新河湾商业中心、潮南嘉盛金光广场等商业综合体，粤东物流产业园、宝奥城、广澳物流园、国美物流城等现代物流园区，粤东家居建材博览中心、粤东（国际）电子交易中心、国瑞会议会展中心等一批专业市场、会展中心建设。推动都市型电子商务和农村电子商务双向发展，加快柏亚电商大厦、宝奥电商大楼、邮政跨境电商产业园等项目建设；推进县域电子商务工作，与阿里巴巴、360、京东等大型电商集团合作，促成“淘宝·特色中国”之汕头馆、潮汕馆等项目落地运营；积极探索电商新业态，支持企业发展线上线下、双线并进的电商模式。大力发展现代旅游业，加快内海湾旅游规划，推进南澳国际高端旅游休闲岛、潮人码头、国际客运码头邮轮项目、南山湾中信酒店、海门莲花峰风景区、前美古村潮侨文化旅游区、锦峰动感世界、雷岭温泉度假村等重点项目建设。积极创建“版权兴业示范基地”，培育发展影视制作、动漫游戏等文化创意产业。

推动传统优势产业高端化。搭建产学研合作平台，推动传统优势产业企业与高校、科研院所共建研究开发院，吸引高校、科研机构、大型企业在汕建立分支机构，加快研发、设计、检测等公共科技创新平台建设，实施数控一代机械产品创新应用示范工程。加大工业技改投资，实施工业转型升级攻坚三年行动计划，激发民间技改动力，实施智能化改造和设备更新，推动企业转型升级。加快国药集团生物医药产业、广药和西门子全自动酶免分析系统等项目落地建设，推动生物医药制药、精细化工聚集升级。加快建设国家玩具质检中心、日用精细化工国家质检中心和包装机械、文具省质检站等一批公共技术服务平台，积极创建国家级出口玩具安全质量示范区、中国潮南纺织产业创新基地，为传统产业改造提升提供技术支持。紧紧抓住广东以色列理工学院落户汕头的机遇，探索合作创办中国以色列创新产业城，打造区域高层次创新基地、产业孵化基地、国际科技合作基地和国内外人才聚集基地。大力发展海洋产业，支持南澳建设“国家级海洋生态文明建设示范区”和“广东省海洋综合开发试验县”。

促进园区扩能增效。加大园区二次开发力度，支持园区构建投融资平台，鼓励民间资本参与园区建设。加快建设一批专业特色园区，依托现有产业优势，整合原有小园区，规划建设金平生物医药产业园、龙湖龙东产业园、澄海春天湖循环经济产业基地、潮阳针织内衣工业园和潮南家居服工业园等园区。充分发挥高新区优势，重点扶持孵化一批科技创新型项目和企业增资扩产。争取尽快获批保税物流中心（B型），复制推广上海自贸区14项监管制度，推进保税区升级转型。加快建设一批设施农业、畜禽生态健康养殖、休闲生态农业、农产品物流等现代化园区。

（四）加快推进城市扩容提质

以华侨试验区为引擎，加快推进城市扩容提质，打造绿色、低碳、智慧城市，努力提升汕头作为区域中心城市的吸引力和承载力。

全面启动华侨试验区规划建设。完成试验区总体规划及基础设施、产业引进等专项规划编制工作。加快起步区各功能组团建设。东海岸新城重点是加快市政道路、桥梁等基础设施建设，加大招商引资和土地营销策划力度，推动一批现代产业项目落地。南滨新城重点是加快推进中轴线景观工程、岸线景观工程建设，配套完善市政基础设施。珠港新城重点是加快市政基础设施和总部经济园区建设，推动一批企业总部和现代服务业项目落地。整合广澳港、保税区资源，推进区港联动，做大做强南部临港经济。

推动城乡综合运营开发。创新城乡发展投融资机制，大力推广PPP模式，策划包装一批项目，面向社会资本招商，撬动和推进一批重大基础设施和产业项目建设。加快西部生态智慧新城土地一级开发和金凤西路、西港河桥、金砂西路、澄海路及大学路立交等路网基础设施建设。在月浦片区规划建设金平现代特色产业园，重点建设轻工装备城、生物制药城、印刷包装城和食品城，促进产城融合，打造汕头北部新的城市组团。推进粤东物流总部新城、潮阳东部新城、潮阳练江新城、潮南行政新区、厦深高铁潮阳站（谷饶）物流新城等一批项目开发建设。推进节约集约用地及城乡土地生态利用制度综合改革试点工作，加大闲置低效用地整合、收储、处置力度，保障土地有效供应，促进土地节约集约使用。

继续推动城市更新。把信息化摆上重要战略任务，加快智慧城市建设，推进城市基础设施智能化。着重发展第四代移动通信，推进城市百兆光纤工程和宽带乡村工程，推进宽带提速普及和WiFi热点广覆盖。推进城市改造更新，加快中山路、长江路、长平路等一批破损道路、“水浸街”改造，打通金环南路等“断头路”。保护提升老城区，保育活化开埠历史文化街区，建成开放永平路示范项目。加快推进西港、乌桥岛和光华片区旧城改造工程。

（五）着力营造平安有序和谐的人居环境

在加快汕头振兴发展的同时，要下大力气加强城市综合管理和生态环境保护，维护社会平安、和谐、有序，为人民群众生产生活营造良好环境。

加强城市综合管理。加大市容环境整治力度，推进街区形象提升工程、小贩疏导工程、集贸市场升级改造工程。开展城市管理联合执法，集中公安、城管、交通、工商部门执法力量，重拳整治各种城市管理乱象。推进道路交通秩序整治，坚决查处各种交通违法行为，突出整治摩托车失管失序、三轮车“卷土重来”、出租车不规范经营问题。在此基础上，建立健全城市管理联合执法长效机制，从根本上破解城市管理“治乱循环”难题。实施《中心城区核心片区交通改善规划》，改造提升交通微循环系统，优化交通流组织设计，加强道路交通智能化建设，缓解交通拥堵问题。

维护社会平安和谐。深化“平安汕头”创建，落实市委关于“社会矛盾化解攻坚年”工作部署，继续开展“三类重点地区整治”，着力打造群防群治的社会治安立体防控体系，不断提升群众安全感。进一步完善重大决策社会稳定风险评估、维稳和治安形势分析、矛盾纠纷排查化解和应急处置工作机制，切实维护社会大局稳定。深入开展军政军民共建活动，巩固发展“全国双拥模范城”创建成果。加快推进农村集体“三资”管理服务平台建设，夯实农村基层民主自治的基础。坚持抓安全生产与抓经济发展同部署、同实施，落实“党政同责、一岗双责、齐抓共管”要求，建立健全日常检查监督制度和隐患排查治理长效机制，努力实现安全生产形势持续好转。加强市场监管，加大打击假冒伪劣商品力度。完善食品药品监管机制，加快安全追溯体系建设。

持之以恒推进贵屿和练江流域综合整治。强力推进贵屿环境污染整治，全天候全覆盖严厉打击污染违法行为。按照“统一规划、统一建设、统一运营、统一治污、统一监管”的要求，加快推进贵屿循环经济产业园区建设，确保集中拆解楼、湿法冶炼、火法冶炼等园区内生产服务性项目按期投入使用，拆解户集中进园生产，完成省下达的挂牌整治目标任务。继续推进北港河底泥、贵屿重金属污染、龙港渡头基本农田等修复示范工程。全力推进练江流域综合整治。动工建设练江干流陇田段、海门堤围，潮南区新坛、锣鼓陂水闸和潮阳区北港、后溪水闸；加快铜盂、和平、陈店、司马浦、陇田等污水处理厂及配套管网建设，启动纺织工业循环经济产业园建设。

加强生态环境建设。开展国家环境保护模范城市创建工作。加强环保基础设施建设，确保列入国家减排责任书项目的7个污水处理厂按期建成投入使用。加快雷打石生活垃圾卫生填埋场扩建工程、雷打石环保电厂和潮阳、潮南区生活垃圾焚烧发电厂建设，配套完善各镇（街道）垃圾转运站建设。深入推进重污染行业整治，完成万丰热电厂整改任务。扎实推进“美丽乡村、幸福村居”创建活动，积极创建宜居社区、村庄、城镇。加强水利基础设施建设，推进“千里海堤”、病险水库、水闸除险加固。

（六）统筹推进城乡基本公共服务均等化

以更大的投入和更实的举措，加快发展教育、文化、卫生、体育等社会事业，统筹推进城乡基本公共服务均等化，让人民群众共享改革发展成果。

稳定和扩大就业。支持鼓励以创业带动就业，抓好公共就业服务标准化、信息化建设。今年新增城镇就业6万人，农村劳动力技能培训2．25万人，农村劳动力转移就业5万人。

着力推动教育规模和内涵协调发展。推进教育创强和义务教育均衡发展，做好潮阳、潮南10个镇（街）的教育强镇（街）和濠江、潮阳、潮南三个区的教育强区以及全市教育强市的申报工作，继续开展创建全国义务教育基本均衡发展区（县）工作，加快义务教育标准化学校建设全覆盖，做好第二批14所薄弱普通高中项目学校建设工作。启动学前教育第二个三年行动计划，每个乡镇都建设一所公办规范化乡镇中心幼儿园，提高农村学前教育水平。加快中等职业教育发展，办好汕头职业技术学院，推进市高级技工学校创建技师学院。支持汕头大学、粤东高级技工学校办学。积极协助筹办广东以色列理工学院，基本完成北校区建设，启动招生与教学准备工作。实施特殊教育提升计划，让每一个残疾孩子都有机会接受合适的教育。规范发展特色民办教育，促进多元化办学。推进教师队伍建设，鼓励高校毕业生到农村任教，提升教学质量和科研水平。推进终身教育体系和学习型社会建设。

切实改善医疗卫生公共服务。深化医药卫生体制改革，统筹推进医疗保障、医疗服务、公共卫生、药品供应、监管体制综合改革。加快推进汕大医学院附属肿瘤医院、市中医院、市妇幼保健院、市皮肤医院、汕大国际眼科中心异地建设，加快建设白求恩潮阳医院和汕头国瑞医院、东方医院，支持汕大医学院建成粤东医教、科研和民众健康教育推广中心，强化粤东医疗卫生服务中心地位。开展镇卫生院标准化建设，提升基层医疗机构服务能力。建设市级区域医疗卫生信息平台，提高医疗卫生信息化水平。深入开展优生优育工作和计生综合整治，提升人口计生工作水平。深化殡葬改革，推广绿色生态殡葬。

加快文化体育事业发展。推进潮汕历史文化博览中心、新档案馆等新建重大文化场馆的建设及布展工作，改造升级一批农家书屋。加强文物、非物质文化遗产、记忆遗产的保护和利用，全面推进“海上丝绸之路”申遗工作。加快全民健身公共服务体系建设，深入开展全民健身活动。

完善多层次社会保障体系。扩大社会保障覆盖面，确保全市基本社会保险覆盖率达到97％，其中城镇职工养老保险覆盖率达到95％，城乡居民养老保险覆盖率达到99％，基本医疗保险覆盖率达到98％。加快发展养老服务业，继续实施普惠型高龄津贴制度和银龄安康行动，加快推进养老床位建设，各类养老床位达到1．6万张。支持社会力量举办养老机构，推广邻里互助养老新模式。扩大社会福利普惠面，促进社会慈善事业发展，完善社会救助体系。

（七）继续办好十大民生实事

今年市级财政将投入10．53亿元，继续办好十大民生实事。

一是实施市容市貌提升工程。在金平区、龙湖区推行环卫作业市场化保洁；开展城市管理联合执法，整治城市路面秩序；加快淘汰“黄标车”；扩建雷打石生活垃圾卫生填埋场。

二是加大公共交通便民利民力度。新增投放100部环保公交车；实施老年人和学生乘坐公交车补贴政策；建设智能化公共交通体系。

三是提高底线民生保障水平。基础养老金财政补助月人均提高为95元，7月1日起补助标准提高为100元；城乡低保月人均补差水平提高到374元和172元；农村五保供养标准不低于上年度当地农村居民人均纯收入的70％；孤儿集中供养和散居供养标准分别提高到月人均1240元和760元；城镇“三无”人员集中供养基本生活费标准月人均1000元，社会散居供养月人均800元。

四是实施教育普惠工程。继续提高城乡免费义务教育公用经费标准，小学和初中分别提高到每生每学年1150元和1950元；山区和边远农村教师补贴提高到人均每月不少于700元；扩大中等职业教育免学费政策范围，完善助学金制度。

五是提高医疗卫生保障和服务水平。城乡居民医保补助标准提高为每人每年380元；实施农村已离岗接生员和赤脚医生生活困难补助；为全市25所一般乡镇卫生院、8所中心卫生院配置“五个一”设备；实施边远乡镇卫生医务人员岗位津贴，对乡镇卫生院在编人员按照初级以上职称、执业助理医师资格（注册护士）、其他人员分别按月人均650元、500元、400元的标准发放岗位津贴。

六是加大残疾人救助力度。为1500人次贫困重性精神病患者提供住院医疗救助；为600名7周岁以下残疾儿童提供一年康复训练补贴；为700名贫困白内障患者免费实施复明手术；为贫困残疾人适配辅助器具1500件；贫困残疾人生活津贴和重度残疾人护理补贴分别提高为每人每年1200元和1800元；为1800名长期卧床的特困（低保）残疾人提供每人一年1200元的家居护理或一次性医疗补助；为500人次听障人士提供听力语言康复救助。

七是实施文化体育惠民工程。完成100个农家书屋达标升级，建设配套10个社区文化广场；在全市541个行政村每村每月放映一场公益电影；免费开放博物馆（纪念馆）、公共图书馆、文化馆、文化站；每周五晚在慧如剧场开展“潮剧大观园——周五有戏”免费及低票价惠民演出；每月组织两次潮剧潮艺进社区、进农村、进企业惠民活动；新增100所中小学校体育设施向社会开放；加强群众体育活动设施建设，配套群众体育场所健身器材，免费或低收费开放体育场馆；修缮配套潮汕抗战纪念馆、桂园等历史文物场馆。

八是推进城乡保障性住房建设。筹集公租房2000套，发放租赁住房补贴500户，基本建成公租房2000套，城市棚户区改造1400套。

九是继续实施“绿满家园”全民行动。继续实施“绿满家园”三年计划，重点对儿童公园北侧及泰山路、潮汕路等城市主出入口周边绿化改造；启动一批森林生态项目建设，营造碳汇林2．2万亩，森林抚育面积5．8万亩，建设一批绿化美化示范村；提高市政设施及绿化管养水平。

十是改善农村生产生活环境。继续推进千村环境卫生整治和“百河千沟万渠大整治”行动；完成扶贫开发“双到”任务，增强全市新一轮48个省定贫困村及8个涉渔社区的“造血功能”；继续实施潮阳区、潮南区村村通自来水工程，实现两区行政村村村通自来水覆盖率90％以上、农村自来水普及率90％以上、农村生活饮用水水质合格率均达到90％以上的工作目标。

各位代表！做好2015年经济社会发展各项工作，必须深入推进依法行政，坚持不懈打造阳光法治服务政府。一是坚持依法科学民主决策。自觉接受市人大及其常委会的法律监督、工作监督和市政协的民主监督，认真听取人大代表、政协委员、民主党派、工商联、无党派人士和各人民团体的意见。坚持公众参与、专家论证、风险评估和集体决策机制，落实重大决策合法性审查制度，增强法律审查在政府重大决策中的风险防范功能，确保行政决策的科学性和准确性。二是着力提高依法行政水平。加强和改进政府立法，推动立法和改革决策相衔接，健全政府法律顾问制度。深化行政执法体制改革，推进综合执法，坚持严格规范公正文明执法，健全行政执法和刑事司法衔接机制。严格执行《汕头市行政程序规定》，规范行政执法、行政听证、行政公开等程序，确保行政权力在法治轨道上运行。加强公务员教育管理，运用法治思维和法治方式推动经济社会发展，提升政府工作法治化水平。三是加强对行政权力的制约和监督。健全法律监督、民主监督和舆论监督机制。完善政府内部层级监督，推进“阳光复议工程”。深化政务公开，重点完善基层政务和各领域办事公开制度。健全监察、司法、审计等联动的行政权力监督机制。四是狠抓政风建设。深入落实中央八项规定、“三严三实”等要求，巩固拓展党的群众路线教育实践活动成果，保持整治“四风”高压态势。深入开展政风行风专项整治，继续改进文风会风。大兴求真务实之风，坚持用结果说话，以实干高效的作风取信于民。

各位代表，伴随着华侨试验区的设立，汕头的发展再次上升为国家战略。站在新起点，推进大发展，实现新跨越，是时代赋予我们的历史重任。让我们在省委、省政府和市委的坚强领导下，紧紧依靠全市人民，凝聚海内外潮人的智慧和力量，开拓创新，扎实工作，为开创特区振兴发展、跨越发展新局面而努力奋斗！
